# The New Chemical Notation

**Published:** 1877-08

**Authors:** J. H. Logan

**Affiliations:** Atlanta, Ga.


### THE NEW CHEMICAL NOTATION.

BY J. H. LOGAN, A.M., M.D., ATLANTA, GA.

In our short paper on Chemical Nomenclature, the irresponsible types saw proper to represent us in one place, at least, as writing what we did not mean to write, and we must take this opportunity to correct it. On page 152 we did not say: "They are carbonic dioxide and carbonic ACIDS," but they are carbonic dioxide and carbonic oxide—the only known compounds of carbon and oxygen.

In the same paper, we promised to show secondly, that where the system of nomenclature fails, or falls short of its design, its place is supplied by a still more comprehensive and admirable system of notation; and that, whenever this appears to present perplexing difficulties, they may be traced partly to the impatience of prejudice, and partly to the abstruce nature of the subject embraced in the discoveries of a rapidly advancing science.

In the notation—the new notation—of chemistry there is an argument for the existence of atoms and molecules, intimates Prof. Cook, in the very beauty of its admirable power to express their mysterious chemical union and products. It reminds one vividly of the presumptive argument for the undulatory theory of light, found from the analogy of air vibrations in the possibilities of sound.

It was in 1815 that the celebrated Berzelius first suggested the idea of symbols for the representation of atoms and molecules in chemical elements and compounds, little dreaming, doubtless, like the great Genoese navigator, of the real extent and importance of the invention his genius was conferring upon the world. Yet, so unpretentious and transparent is the entire system, that in its very simplicity we are in danger of losing sight of the real greatness of its conception, and that it is worthy of the admiration of sages, as well as of children. Besides simplicity, familiarity too, often blunts the perception of worth, and we know but one thing in all the world of science, to which, in this regard, we can compare this notation of chemistry, and, as it should be for a sound argument, it is something of the same kind—it is the Arabic notation, which, indeed, underlies the whole system of mathematics. We challenge any man to point out a more seemingly childish, and yet grander thing in reality than that notation. It is associated with all that is joyous or unhappy in schoolboy days, and is the very warp of the highest mathematical reasoning of our ripest manhood. Truant history withholds the name of the man whose genius was capable of such scientific devising, but this civilization and the ideal civilization of the future, will, with one consent, could his name be certainly known, erect to his memory a monument more enduring than brass.

Did you ever pause, for one moment, to think, in your almost hourly use of this simple invention, how a child can use it—can amuse or serve himself with it—can understand it, and man can transact with it all that is necessary in the most varied and complex commercial relations, and yet the child, by a few strokes of his pencil, can express with its characters and combinations, what neither himself, nor an Aristotle has intellectual power to grasp? This is one of the essential characteristics of the sublimity of the things we know to be sublime. It is true of great books—of the Bible, Pilgrim's Progress and of Milton's immortal epic. It is true of sublime buildings, and mountains, and waterfalls, and equally of sublime men—of Moses and Luther, and the great Apostle to the Gentiles.

These remarkable facts illustrate what is substantially true of chemical notation, though in a more limited field. Nothing, it will be presently seen, is simpler in its contrivance, and yet few things are more comprehensive, or expansive, in practical applications, than it. It enables us to write, with facility, all we desire to preserve, or study, or transmit, and what a child can read and understand in the science; and, with equal ease, its unlimited symbolical power of expression transcends the capabilities of the mind, to follow it into what it plainly intimates, a mysterious and fearfully potent molecular motion and arrangement. In other words, in chemistry, as in Arabic decimals, we can write with the utmost dispatch, what we cannot, with any dispatch, either express in language, or at all comprehend. The written expression of number has no limit but the intellectual incapacity to understand and give it utterance.

What, then, is chemical notation? It may be thus defined: Notation in chemistry is a system which provides, by means of certain symbols, signs and numerals, properly written, either alone or in conjunction, for the expression in brief, of any element, compound, or reaction known to chemistry. The elemental symbols have been already given and explained in the article on Nomenclature; it is not necessary to repeat them here. H is the symbol of hydrogen gas, and H H, the formula for the simple hydrogen molecule. H expresses not only the fact that an atom of hydrogen is present, but the additional fact that its atomic weight is invariably one; not proportionally, but permanently. H H can be written $H_2$, in which 2, as in algebra, may be called an exponent, but it does not express here, as there, a power, but a multiple; it means two atoms of hydrogen, and not the atom multiplied upon itself. HCl is the symbolical formula for the compound molecule of hydrochloric acid. It is compound because it is made of atoms of different kinds, and clearly conveys the information that chemical union has taken place between the hydrogen and chlorine, and, moreover, that the atoms thus combined, are equal in chemical value, or have the same atomicicity, for the one just neutralizes the other. The nomenclature of the molecule is, of course, hydric chloride.

A somewhat more complex molecule is that of water, $H_2 O$; and, simple as it may appear, this water molecule is the border land of difference between the old and new chemistry. Prof. Cook remarks that the difference between the notation of H O and $H_2 O$, is of a type with the whole difference between the old and new schools of chemistry. The two symbols may be regarded as the SHIBBOLETHS of the two systems. An improved notation, therefore, lies at the foundation of the whole superstructure of the modern science.

The symbol H O fairly states the *proportions* with which hydrogen and oxygen unite to form water, but H O is not the formula of the water molecule, for the simple reason that, though sufficiently expressive of the atomic presence of each element, it fails to give the atomic weight of oxygen. It has just been remarked that an essential principle of the new notation is, that the symbol not only designates an atom of each element, but its atomic weight as well.

The old chemistry knew nothing of atomic weights in the proper sense of the term; its numerical equivalents were mere proportions, which could be changed at pleasure. While the English school, chiefly, selected hydrogen as the basis or standard of their system, the Germans made choice of oxygen; in each system of course, the proportions were the same, but the numbers quite different. If atomic weights are no more than so many proportionate numbers, it is obviously a matter of perfect indifference whether the water molecule be written, $H_2 O$, $HO_2$, H O or $H_2 O_2$, for the numerical values given to the letters, as symbols are purely arbitrary. All is now changed; the new chemistry, as the old, has its combining proportions, but those proportions are not necessarily in every case, the atomic weights of elements.

H O symbolizes a fact equally accepted in both systems, viz: that hydrogen and oxygen unite in the proportion of 1 to 8, to form water, for it is identical with the proportion $H_2 O$, oxygen being 16 by weight; and this, and not 8, is this atomic weight of oxygen, hydrogen being 1; that is 16 is the least portion, by weight, of oxygen that will combine with hydrogen, and two atoms of hydrogen and one of oxygen constitute the molecule of water. A volume of steam weighs nine times as much as the same volume of hydrogen, but the molecule, or two volumes of hydrogen is 2, therefore the molecular weight of water is 18, and hence its molecule contains twice as much of each constituent, 2 and 16.

Atomic weights are no longer a mere sliding scale of proportional numbers, like specific gravity, but fixed and permanent quantities; referred to hydrogen they are as fixed and definite as the numbers used to express the absolute weight of matter—they are the "fundamental constants of chemistry." Numerals are, also, frequently prefixed to molecules, and correspond in use to the coeficient of algebra. $H_2 O$ represents one molecule of water; $2 H_2 O$, two molecules, and $6 H_2 O$, six; the coeficient in each case, multiplying the exponents expressed and understood.

Another and more complex formula, is that of alcohol, $C_2 H_6 O$, but with the explanations already given, it is easily understood. It clearly indicates just what chemistry says alcohol is, a molecule composed of two atoms or twenty-four parts by weight of carbon, six atoms or six parts by weight of hydrogen, and one atom, or sixteen parts by weights of oxygen, and the weight of the whole, or its molecular weight to be 46, that is 46 times the weight of an equal volume of hydrogen. This is its notation; its nomenclature requires a little explanation. The name alcohol is purely arbitrary, given it by the alchemists of the ninth century, before chemistry was known. What is its chemical name? The Lavoisierian rule must not be forgotten. The names of compounds should be derived from those of their constituents. Now the alcohol molecule can be divided into two well known compound radicals, $C_2 H_5$ ethyl, and $H O$, hydroxyl, that is, compounds which are capable of all the chemical behavior of simple elements, and hence their name in contradistinction to simple radicals. The molecule may be written, thus $(C_2 H_5)$, $H O$, and read ethyl hydrate just as K H O, and Na H O are potassic and sodic hydrates. It is sufficient to remark that this hydroxyl H O, has a very different meaning from that of the same symbol used in connection with water; H O, expressive of the proportions in which the two gases unite to form water, is quite different from H O as the definite molecule of a possible or impossible substance. It is unbalanced—lacks an atom of hydrogen to become water, and has therefore, no free existence. Graphically it is, H—O—, having one unemployed link: the other radical satisfies that link and forms alcohol.

We remarked that the new doctrines of quantivalence and substitution are intimately associated with notation; this alcohol molecule is a good example of it. You know what quantivalence is; it is the power of an atom to neutralize hydrogen units, or to take their place by substitution. Now it is a curious fact, not yet understood, that many of the elements have a variable quantivalence; but carbons is generally four, indicated by Roman numerals, while hydrogen has always one. The radical ethyl may be again written $\overset{IV}{C_2}\ \overset{V}{H_5}$, or graphically, $\begin{array}{c} H\ \ H \\ |\ \ \ | \\ H\text{-}C\text{-}C,\text{-} \\ |\ \ \ | \\ H\ \ H \end{array}$ showing that all the bonds of the molecule are engaged, but one; so that ethyl is a MONAD radical.

At this point, we may apply the principle either of substitution or of direct union. $C_2 H_4$ is a saturated molecule, since the quantivalence of C, is here dyadic; ethyl can, therefore, be written, $C_2 H_4$ H, and on the addition of hydroxyl, H O, to this, one of whose hydrogen atoms is wanting, direct combination takes place between it, and the isolated hydrogen of ethyl forming water; and the whole molecule becomes $C_2 H_4 H_2$ O or $C_2 H_6$ O, alcohol again. To get the same result by substitution, write the graphic formula of water, H-O-H, and instead of H O's being added to, let ethyl be substituted for one of the atoms of hydrogen, for being a monad radical, it will replace it and give the formula, H-O-$C_2 H_5$, or $C_2 H_6$ O, alcohol once more.

In conclusion, we give the rule for the management of exponents, when atoms of different quantivalence combine. Each atom must, of course, furnish the same number of bonds, and this number is found from the quantivalances. This is the rule: Find the least common multiple of the two quantivalences; divide this number by the quantevalences in succession, and the result in each case, will be the number of atoms for that constituent.

*Examples:* Let $\overset{\text{I}}{\text{H}}$ and $\overset{\text{II}}{\text{O}}$ combine. Find the least common multiple of 1 and 2; this is 2; now divide 2 by 1, the quant ivalence of H, and it gives 2 as the exponent of H: divide the same by 2, the quantivalence of O, and the result is 1 for the exponent of O. Hence the required formula is $H_2 O$.

$\overset{\text{VI}}{\text{S}}$ and $\overset{\text{II}}{\text{O}}$, a hexad and dyad will form $S O_3$; for the least common multiple of the quantivalences is 6, and the results of division respectively, are 1 and 3, hence the formula is as written above.

This, now, is the head and front of chemical notation, and there is no system, of its kind, in all the circle of the sciences, the mathematics excepted, so admirable and complete. Its illustrious author, Berzelins, in its construction, obviously, wise as he was, builded better than he knew; for it has already, since his day, in great part, superseded the nomenclature, itself scarcely surpassed as a system, and is, at this moment, in the hands of the most distinguished chemists of the world, powerfully aiding in the advancement of the science to new fields of investigation and discovery.

