# Erratum

**Published:** 1877-08

**Authors:** 


Erratum.—On page 269, 13th line from top, "expensive" should read "*explosive*," our proof-reader never having heard of quinine exploding.

